# Supplementary material for: Effectiveness of an Artificial Intelligence Software for Limb Radiographic Fracture Recognition in an Emergency Department
Source: J Clin Med. 2024 Sep 20;13(18):5575. doi: 10.3390/jcm13185575 (PMC11433213; doi:10.3390/jcm13185575)
Supplement: Supplementary file 1 [file jcm-13-05575-s001.zip › jcm-3179636-SI.pdf]

**Supplementary materials**

S1.The code underlying this work can be found online at *https://github.com/facebookresearch/detectron2*.

S2. List of emergency physicians : C.P, D.N, M.N, T.N, T.N, B.A, C.A, D.B, D.M, M.L, M.M, R.E, R.A, R.A, C.R, D.J, G.A, G.J, H.T, K.N, P.S, S.J

List of radiologists (initials) : G.H ( 10 years of experience ), M.M ( 25 y.e ), J-C.F (25 y.e ), J-P.T ( 25 y.e) , J.V (10 y.e ) , N.E ( 20 y.e ), G.F ( 15 y.e ).
